# Association of N-acetylcysteine use with contrast-induced nephropathy: an umbrella review of meta-analyses of randomized clinical trials

**DOI:** 10.3389/fmed.2023.1235023

**Published:** 2023-09-14

**Authors:** Rong Zhu, Rong Zheng, Bing Deng, Ping Liu, Yiru Wang

**Affiliations:** Longhua Hospital, Shanghai University of Traditional Chinese Medicine, Shanghai, China

**Keywords:** percutaneous coronary intervention, computed tomography, coronary angiography, peripheral angiography, acute kidney injury, ST segment elevation myocardial infarction, serum creatinine

## Abstract

**Background:**

The effectiveness of N-acetylcysteine (NAC) in treating contrast-induced nephropathy (CIN) has been the subject of conflicting meta-analyses, but the strength of the evidence for these correlations between NAC use and CIN has not been measured overall.

**Objective:**

To evaluate the data from randomized clinical studies (RCTs) that examined the relationships between NAC use and CIN in meta-analyses.

**Methods:**

Between the creation of the database and April 2023, searches were made in PubMed, Cochrane Library, EMBASE, and Web of Science. N-acetylcysteine, contrast-induced nephropathy, or contrast-induced renal disease were among the search keywords used, along with terms including systematic review and meta-analysis. The Assessment of Multiple Systematic Reviews, version 2, which assigned grades of extremely low, low, moderate, or high quality to each meta-analysis’s scientific quality, was used to evaluate each meta-analysis. The confidence of the evidence in meta-analyses of RCTs was evaluated using the Grading of Recommendation, Assessment, Development and Evaluations method, with evidence being rated as very low, low, moderate, or high.

**Results:**

In total, 493 records were screened; of those, 46 full-text articles were assessed for eligibility, and 12 articles were selected for evidence synthesis as a result of the screening process. Based on the pooled data, which was graded as moderate-quality evidence, it can be concluded that NAC can decrease CIN (OR 0.72, 95% CI 0.65–0.79, *p* < 0.00001) and blood levels of serum creatinine (MD −0.09, 95% CI −0.17 to −0.01, *p* = 0.03). In spite of this, there were no associations between NAC and dialysis requirement or mortality in these studies.

**Conclusion:**

The results of this umbrella review supported that the renal results were enhanced by NAC. The association was supported by moderate-quality evidence.

**Systematic review registration:**

[https://clinicaltrials.gov/], identifier [CRD42022367811].

## 1. Introduction

Contrast-induced nephropathy (CIN) is an occasional complication in patients undergoing contrast-enhanced imaging procedures such as angiography, computed tomography, and magnetic resonance imaging (1). It is defined as an increase in serum creatinine (SCr) levels of more than 44.2 mol/L (0.5 mg/dL), or 25% above baseline within 48 h of receiving an iodine-based contrast (2). The reported incidence of CIN in the literature varies between 3.3 and 14.5%, but the numbers are inconsistent across studies due to the use of different definitions (3). CIN is associated with prolonged hospital stays, increased morbidity and mortality rates, and higher medical costs (4). Several methods, including the use of N-acetylcysteine (NAC), have been suggested for CIN prevention (5).

NAC, a thiol-containing substance, has been proposed as a potential preventive drug for CIN due to its low cost and high tolerability (6). It is believed to work by reducing reactive oxygen species, lowering oxidative stress, and enhancing renal blood flow, thereby providing renoprotective benefits (7). Furthermore, NAC’s antioxidant and anti-inflammatory properties may have a protective effect against CIN (8). The use of NAC and its association with CIN risk have been investigated in several studies (9, 10). However, the results have been conflicting, with some studies suggesting that NAC has a significant health benefit (11, 12), while others report no effect (10, 13). Moreover, the evidentiary quality and risk of bias in these meta-analyses have not been thoroughly evaluated. Large and high-quality clinical trials are needed before NAC can be widely recommended for this indication (14).

To provide a comprehensive summary of the available data on the relationship between NAC use and CIN, we conducted an umbrella review of meta-analyses of randomized controlled trials (RCTs). The objective of this study was to assess the methodological quality of the included meta-analyses, evaluate the reliability and consistency of their conclusions, and provide suggestions for a more definitive response to the question of whether NAC is an effective preventive treatment for CIN.

## 2. Methods

The protocol for this study was registered at the International Prospective Register of Systematic Reviews (PROSPERO; registration number: CRD42022367811).^1^ The study followed the Preferred Reporting Items for Systematic Reviews and Meta-analyses reporting guideline (15).

### 2.1. Literature search and selection criteria

From the beginning of the databases up to April 2023, we conducted a systematic literature search in PubMed, Cochrane Library, EMBASE, and Web of Science. The search strategy employed a combination of keywords related to “acetylcysteine,” “N-acetylcysteine,” “contrast-induced nephropathy,” “contrast-induced acute kidney injury,” “meta-analysis,” and “randomized controlled trial.” A full list of search terms is available in Supplementary Table S1. Additional relevant studies were identified by manually searching the reference lists of eligible papers. We did not apply any language restrictions.

We included studies that met the following criteria: they were meta-analyses of randomized controlled trials that examined the association between NAC use and contrast-induced nephropathy, with no restrictions on comparators or populations. If multiple meta-analyses were available for the same research topic, we selected the one with the largest dataset, as previously described elsewhere (16, 17). We excluded articles without complete text, reviews, meta-analyses of studies with different study designs, and those without a control group.

Two independent reviewers (R.Zhu. and B.D.) screened the titles and abstracts of the articles, followed by a full-text evaluation of potentially eligible articles. Any discrepancies were resolved through discussion with a third reviewer (P.L.).

### 2.2. Data extraction and assessment of evidence credibility

To ensure the accuracy of the data, two reviewers (R. Zhu. and R. Zheng.) conducted separate data extractions, which were then verified by a third reviewer (Y.R.W.). Both the meta-analyses and individual studies were used as sources for the data. Main outcome was rate of CIN and secondary outcomes were Scr, requirement for dialysis, mortality.

To evaluate the analytical integrity of each meta-analysis, the Assessment of Multiple Systematic Reviews, Version 2 (AMSTAR-2) instrument was used. The AMSTAR-2 includes 16 items that evaluate various domains, such as the comprehensive literature search, duplicate study selection and data extraction, and risk of bias assessment. Each domain is assigned a score of “yes,” “partial,” “no,” or “not applicable,” and the overall quality of the meta-analysis is evaluated as extremely low, low, moderate, or high based on the number of elements fulfilled. The AMSTAR-2 tool provides a standardized and objective method for assessing the quality of systematic reviews and meta-analyses, which can aid in clinical decision-making and guide future research (18).

To determine the certainty of evidence for each association in the meta-analyses of RCTs, the GRADE criteria (Grading of Recommendations, Assessment, Development, and Evaluations) were used. The GRADE criteria assess the quality of evidence and generate clinical practice recommendations across five domains, including bias risk, inconsistency, indirectness, imprecision, and publishing bias. GRADEpro version 3.6.1 was used to categorize the level of evidence as very low, low, moderate, or high (McMaster University). When making recommendations, GRADE considers the balance of benefits and harms, patient values and preferences, financial implications, and feasibility of implementation (19).

### 2.3. Statistical analysis

The meta-analyses were replicated independently for RCTs using random-effects models to extract effect sizes of individual studies included in each meta-analysis based on study design for each link between CIN and NAC. Measurement data were expressed as mean and mean deviation (MD) or standard mean deviation (SMD), and count data were expressed as OR or RR. Regarding the methodological (methodology of included studies) and clinical (clinical characteristics of the participants) heterogeneity, we evaluated as not homogeneous due to different intervention periods and methods, and various countries of subjects. The I^2^ statistic was used to evaluate the degree of methodological variability (20). Based on these, random-effect model was used to perform the analysis. The threshold of significance for all tests was fixed at 2-sided *p* = 0.05. For statistical studies, Review Manager (Version 5.3) was used.

### 2.4. Subgroup analysis and sensitivity analysis

Where sample sizes permitted, subgroup analyses were performed by count data expressed as odds ratio (OR) or relative risk (RR). We performed several sensitivity analyses to guarantee the robustness of the correlations that were originally rated as having strong or middling evidence. To begin, we used a method that deleted papers with a high risk of bias. Furthermore, we deleted small studies (25th percentile) and main studies with a high risk of bias or low-quality evidence, according to the standards described in reference (21). Additionally, we conducted an approach that excluded studies with high risk of bias. These sensitivity studies were carried out to confirm the robustness and consistency of the original results.

## 3. Results

### 3.1. Study selection and study characteristics

**Figure 1:**
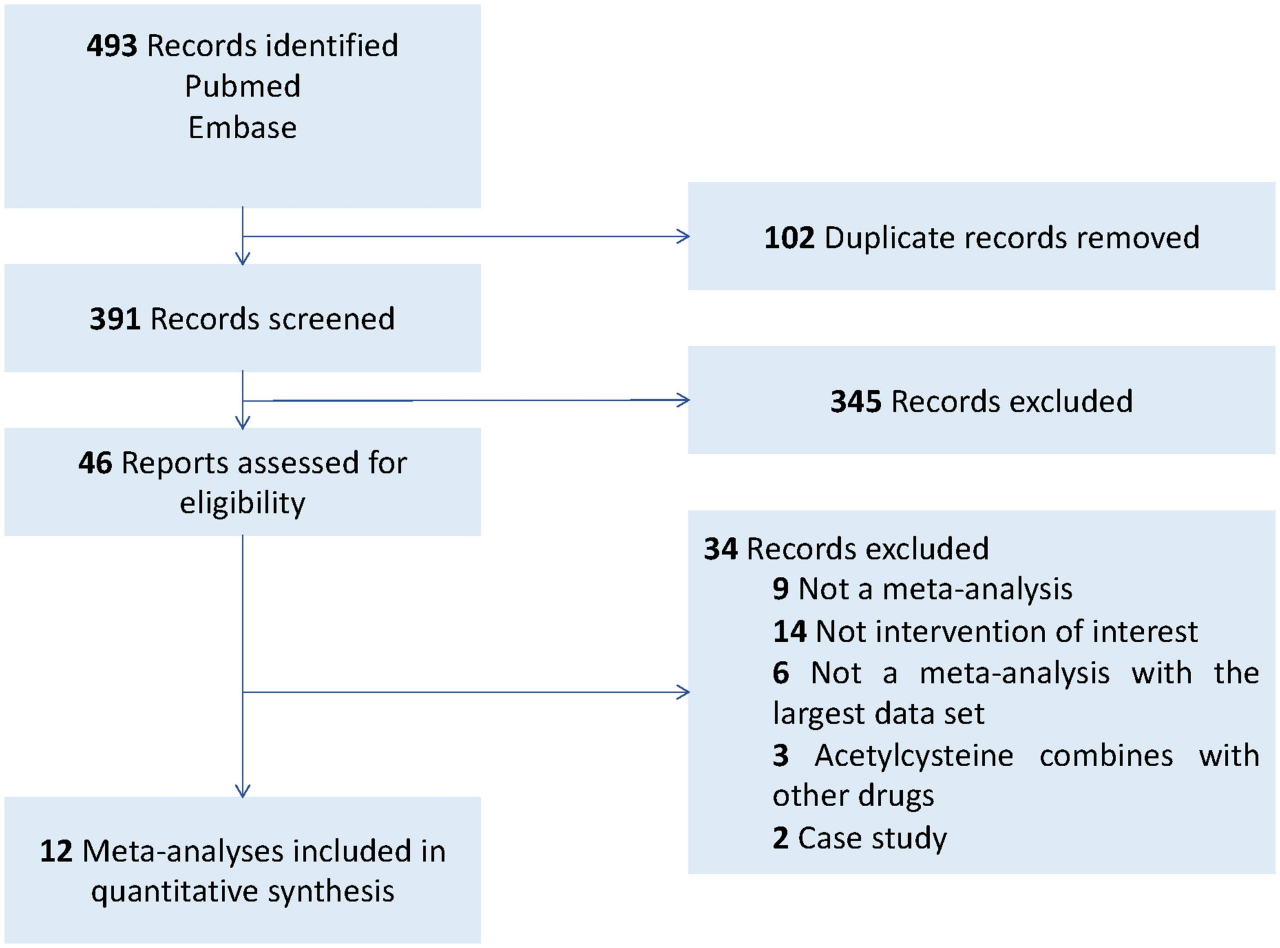
Flow diagram illustrating literature search and study selection.

The process of study selection is presented in Figure 1 and Supplementary Table S1. A total of 493 articles were identified through a systematic search in the electronic databases from the beginning of the databases until April 2023. After removing 102 duplicates, 391 articles were screened by reviewing their titles and abstracts. Of these, 46 articles were considered for full-text review. Ultimately, 12 meta-analyses (22–33) were included in our overall meta-analysis. A summary of the excluded papers after following the selection criteria for overlapping meta-analyses is provided in Supplementary Table S2.

The studies included in this meta-analysis were conducted from 2004 to 2022 in various countries, including China (23, 25, 30–32), Canada (22, 27), the United States (24, 26, 28, 33), and Australia (29). The number of RCTs included in each meta-analysis ranged from 6 to 101, with a total sample size ranging from 485 to 18,729 individuals. Contrast substances were used in a variety of procedures, such as computed tomography, left cardiac catheterization, percutaneous coronary intervention, peripheral angiography, and coronary angiography. Table 1 presents the details of the included meta-analyses.

**Table 1:** Characteristics of meta-analyses of randomized clinical trials.

Source	Search date	Population	Intervention	CON	Outcomes	No. of studies	Participants	AMSTAR-2 rating
Bagshaw 2004	June 2004	CIN patients undergoing intravascular angiography	NAC plus hydration	Hydration alone	CIN incidence, change in Scr, requirement for dialysis	14	1,365	7 Low
Feng 2018	February 2017	CIN patients undergoing contrast administration	NAC	Ascorbic acid	CIN incidence, change in Scr, requirement for dialysis, mortality, length of hospital stay, length of intensive care unit stay	6	919	11 Moderate
Gonzales 2007	March 2007	CIN (CT, LHC, PCI, PA)	NAC	Saline intravenously	CIN incidence, requirement for dialysis, change in Scr, mortality	22	2,746	9 Moderate
Li 2017	January 2015	CIN patients undergoing coronary angioplasty	NAC	Hydration	CIN incidence, change in Scr, requirement for dialysis, mortality, length of hospitalization	19	4,514	11 Moderate
Loomba 2016	None	CIN patients undergoing peripheral vascular or coronary angiography	NAC	Placebo	CIN incidence, requirement for dialysis, all-cause mortality, change in Scr	27	5,555	12 High
Magner 2022	January 2020	CIN	NAC	Placebo, NS+ placebo, NaHCO_3_, theophylline, fenoldopam, or NS+ statin	CIN incidence, kidney replacement, mortality, length of hospitalization	101	32,235	15 High
Trivedi 2009	February 2008	CIN	NAC	Hydration or placebo	CIN incidence, requirement for dialysis, change in Scr, length of hospital stay	16	1,677	5 Low
Wang 2016	October 2015	CIN patients undergoing CAG with or without PCI	NAC	Placebo	CIN incidence	43	3,277	11 Moderate
Wu 2013	October 2012	CIN patients undergoing contrast enhanced CT	NAC	Hydration alone	CIN incidence, change in Scr and cystatin C, requirement for dialysis	6	485	8 Moderate
Xie 2021	March 2020	CIN	NAC	Without NAC	CIN incidence, change in Scr	58	18,729	9 Moderate
Xu 2016	January 2016	CIN	NAC	Hydration, hydration+ placebo or without NAC	CIN incidence	66	11,480	12 High
Zagler 2006	November 2003	CIN	NAC + hydration	hydration alone	CIN incidence	13	1892	7 Low

CT, computed tomography; LHC, left heart catheterization; PCI, percutaneous coronary intervention; PA, peripheral angiography; ST segment elevation myocardial infarction (STEMI); PPCI, primary percutaneous coronary intervention; NAC, N-acetylcysteine; NS, saline solution; CAG, coronary angiography.

### 3.2. Methodological quality and sensitivity analysis

After applying the AMSTAR-2 tool to assess the methodological quality of 13 meta-analyses, we observed that three of them (23.1%) had high methodological quality, seven meta-analyses (53.8%) had moderate methodological quality, and the remaining three meta-analyses (23.1%) had critically low methodological quality (refer to Table 1). We summarized the GRADE results for quality assessment in Table 2 and Supplementary Table S3, indicating that the level of confidence in the findings of the included literature was low, moderate, and high in 21.1, 52.6, and 26.3% of the systematic reviews, respectively. In addition, excluding RCTs with small size or removing RCTs with a high risk of bias, associations initially retained the same rank (Supplementary Table S4).

**Table 2:** Summary of associations of NAC and outcomes.

Source	Population	Intervention	Control group	No. of studies	No. of Participants	GRADE rating	AMSTAR-2 rating
CIN incidence							
Bagshaw 2004	CIN patients undergoing intravascular angiography	NAC plus hydration	Hydration alone	14	1,365	High	7 Low
Feng 2018	CIN patients undergoing contrast administration	NAC	Ascorbic acid	3	683	Moderate	11 Moderate
Gonzales 2007	CIN (CT, LHC, PCI, PA)	NAC	Saline intravenously	22	2,746	Low	9 Moderate
Li 2017	CIN patients undergoing coronary angioplasty	NAC	Hydration	19	4,514	Moderate	11 Moderate
Loomba 2016	CIN patients undergoing peripheral vascular or coronary angiography	NAC	Placebo	23	5,199	Moderate	12 High
Magner 2022	CIN	NAC	Placebo, NS+ placebo, NaHCO3, theophylline, fenoldopam, or NS+ statin	101	32,235	Moderate	15 High
Trivedi 2009	CIN	NAC	Hydration or placebo	16	1,677	Moderate	5 Low
Wang 2016	CIN patients undergoing CAG with or without PCI	NAC	Placebo	43	6,554	Moderate	11 Moderate
Wu 2013	CIN patients undergoing contrast enhanced CT	NAC	Hydration alone	6	496	Low	8 Moderate
Xie 2021	CIN	NAC	Without NAC	57	18,592	Moderate	9 Moderate
Xu 2016	CIN	NAC	Hydration, hydration+ placebo or without NAC	66	11,481	Moderate	12 High
Zagler 2006	CIN	NAC + hydration	hydration alone	13	1892	High	7 Low
Change in Scr							
Bagshaw 2004	CIN patients undergoing intravascular angiography	NAC plus hydration	Hydration alone	8	623	High	7 Low
Feng 2018	CIN patients undergoing contrast administration	NAC	Ascorbic acid	4	663	High	11 Moderate
Loomba 2016	CIN patients undergoing peripheral vascular or coronary angiography	NAC	Placebo	16	1868	Low	12 High
Wu 2013	CIN patients undergoing contrast enhanced CT	NAC	Hydration alone	4	233	Low	8 Moderate
Xie 2021	CIN	NAC	Without NAC	11	1,318	Moderate	9 Moderate
Requirement for dialysis							
Gonzales 2007	CIN (CT, LHC, PCI, PA)	NAC	Saline intravenously	22	2,746	Low	9 Moderate
Loomba 2016	CIN patients undergoing peripheral vascular or coronary angiography	NAC	Placebo	7	3,585	Moderate	12 High
Mortality							
Loomba 2016	CIN patients undergoing peripheral vascular or coronary angiography	NAC	Placebo	3	453	High	12 High

CT, computed tomography; LHC, left heart catheterization; PCI, percutaneous coronary intervention; PA, peripheral angiography; ST segment elevation myocardial infarction (STEMI); PPCI, primary percutaneous coronary intervention; NAC, N-acetylcysteine; NS, saline solution; CAG, coronary angiography.

### 3.3. Effect of NAC on CIN

**Figure 2:**
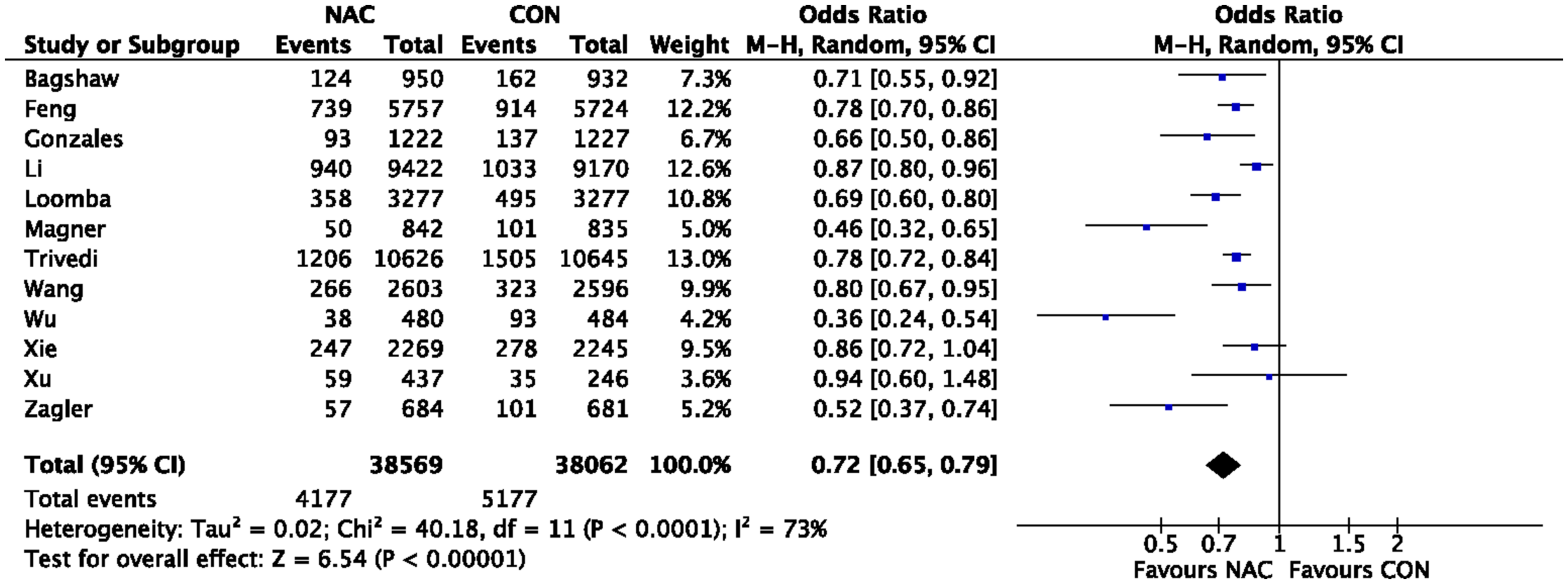
Meta-analysis of studies examining the effect of NAC on CIN outcome. 95% CI, 95% confidence interval; IV, inverse variance.

**Figure 3:**
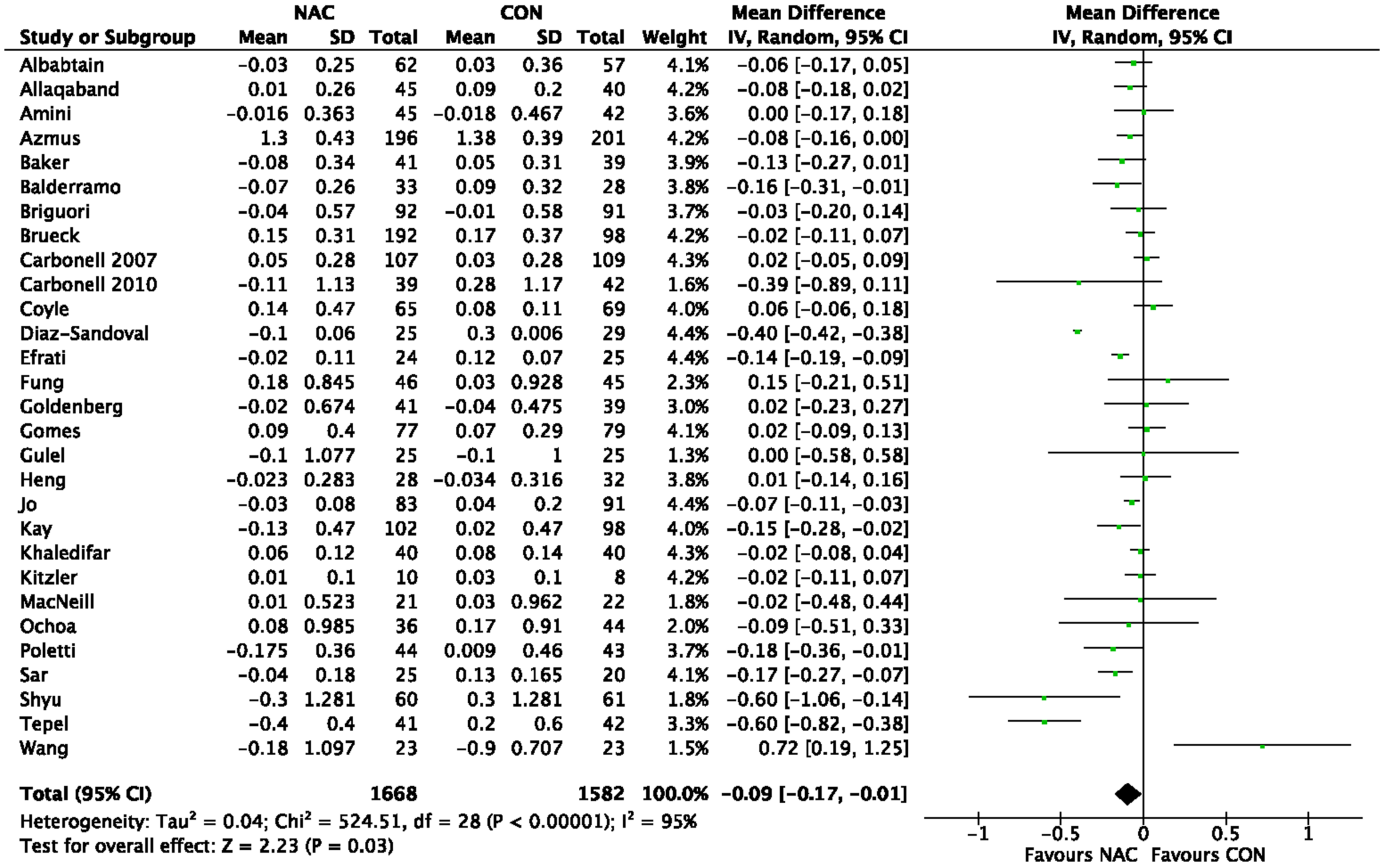
Meta-analysis of studies examining the effect of NAC on Scr Levels. 95% CI, 95% confidence interval; IV, inverse variance.

A total of 38,053 individuals from 161 RCTs were included in 12 meta-analyses (22–33) that explored the influence of NAC on CIN overall. Our pooled effect size showed a significant effect (OR 0.72, 95% CI 0.65 to 0.79, *p* < 0.00001) (Figure 2). Additionally, the funnel plot used in visual examination to demonstrate publishing bias (Supplementary Figure S1). As a consequence, we combined all of the RCT results from each meta-analysis and divided them into groups based on OR or RR. A significant impact was also seen in the subgroup analysis of OR or RR (OR 0.76, 95% CI 0.68 to 0.86, *p* < 0.00001; RR 0.88, 95% CI 0.83 to 0.93, *p* < 0.0001) (Supplementary Figure S2; Figure 3). The evaluation for CIN proof quality was moderate (Table 2).

### 3.4. Effect of NAC on Scr levels

A total of 3,250 individuals from 29 RCTs were included in five meta-analyses (22, 23, 26, 30, 31) that explored the influence of NAC on Scr levels. Our pooled effect size showed a significant effect (MD −0.09, 95% CI −0.17 to −0.01, *p* = 0.03) (Figure 3). Furthermore, the funnel plot shows a minimal danger of publishing prejudice (Supplementary Figure S4). Scr levels’ proof quality was rated as moderate (Table 2).

### 3.5. Effect of NAC on requirement for dialysis

**Figure 4:**
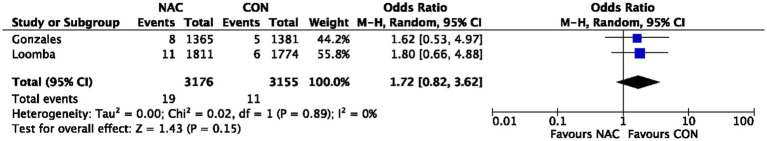
Meta-analysis of studies examining the effect of NAC on Requirement for Dialysis. 95% CI, 95% confidence interval; IV, inverse variance.

The influence of NAC on the need for dialysis was examined in two meta-analyses (24, 26), but our combined effect size found no significant effect (OR 1.72, 95% CI 0.82–3.62, *p* = 0.15) (Figure 4). The level of proof supporting the need for dialysis was rated as high (Table 2).

### 3.6. Effect of NAC on mortality

NAC’s influence on mortality was only examined by one meta-analysis (26), which included 3,585 individuals from 7 RCTs. The pooled effect size showed a significant effect (OR 0.85, 95% CI 0.56–1.29, *p* = 0.45). CIN’s proof quality received a high rating (Table 2).

## 4. Discussion

CIN is a frequent complication of contrast-enhanced imaging procedures and is linked to increased morbidity and mortality (3, 4). NAC has been proposed as a possible preventative drug for CIN, but the quality of supporting data remains uncertain. This comprehensive analysis of meta-analyses of RCTs aimed to evaluate the overall quality and coherence of the evidence supporting the link between NAC use and the prevention of CIN.

Our study revealed that the risk of CIN and Scr levels are significantly decreased with NAC use, which is consistent with earlier findings from meta-analyses and systematic studies (27–31). Our results are in line with the most recent recommendations that high-risk patients undergoing contrast-enhanced procedures could take NAC as an add-on therapy to avoid CIN (34). However, further research is necessary to determine the optimal dosage, duration, and timing of NAC therapy. By combining the findings of numerous meta-analyses that examined the relationship between NAC and CIN across various populations and contexts, our research offers a more comprehensive analysis of the data. Our sensitivity analysis, which excluded studies with poor quality or small sample sizes, had no significant impact on the overall results.

However, our study did not find any evidence of a substantial impact of NAC on the need for dialysis or mortality. This is consistent with earlier studies that found no appreciable change in the need for dialysis or mortality with NAC therapy (24, 26). The heterogeneity observed in the meta-analyses, which may reflect variations in research populations, treatments, and outcomes, could also contribute to the absence of association. The I^2^ statistic was used to measure methodology heterogeneity, and some of the meta-analyses had intermediate to high levels of heterogeneity. Future research should explore possible sources of variability and impact modifiers to better understand the relationship between NAC use and CIN prevention.

This research has some limitations. First, since this was an umbrella review, the quality of the included meta-analyses and their primary studies was critical. The GRADE and AMSTAR-2 tools were used to assess the methodological quality of the meta-analyses, but we were unable to rate the quality of individual studies included in the meta-analyses. Second, it is possible that overlapping primary studies were used in the meta-analyses included in our analysis, which could have introduced bias and impacted the final findings. However, applying selection standards for overlapping meta-analyses and excluding papers that did not adhere to them helped to address this limitation. Third, our analysis was restricted to RCTs, which may not accurately represent how successful NAC use is in preventing CIN in real-world settings. Fourth, we were unable to perform subgroup analyses by dose or time since this was an umbrella meta-analysis based on the meta-analysis, which meant that the data was incomplete.

Additionally, it is important to note that NAC itself is not risk-free. NAC is not recommended for allergy, asthma, severe airway obstruction, or severe respiratory failure in elderly patients. If a clinician uses NAC prophylactically to treat CIN, it is important to make decisions based on the situation of individual patient.

## 5. Conclusion

In conclusion, our comprehensive analysis of meta-analyses of RCTs suggests a significant association between NAC use and a decreased risk of CIN. Our study incorporates multiple meta-analyses and examines sources of heterogeneity and inconsistency to provide a more comprehensive evaluation of the evidence. While our findings are consistent with those of previous meta-analyses and systematic reviews, further research is needed to determine the optimal dose, duration, and timing of NAC therapy for CIN prevention and treatment.

## Data availability statement

The original contributions presented in the study are included in the article/Supplementary material, further inquiries can be directed to the corresponding authors.

## Author contributions

YW and PL developed the review question and designed the research. RZhu and RZhe conducted the publication search, study selection, data extraction, and quality appraisal. BD analyzed the data. RZhu, RZhe, and BD wrote the first draft of the manuscript. YW and PL critically revised the manuscript and contributed to the final version. All authors interpreted the data, read the manuscript, and approved the final version.

## Funding

This work was supported by the National Natural Science Foundation of China (grant nos. 81873245 and 82204849), Training Program for High-caliber Talents of Clinical Research at Affiliated Hospitals of SHUTCM (grant no. 2023LCRC01), Traditional Chinese Medicine Research Project of Shanghai Municipal Health Commission (grant no. 2022QN056), Regional Medical Centre of Longhua Hospital Affiliated to Shanghai University of Traditional Chinese Medicine (grant no. ZYZK001-029), and Clinical Technology Innovation Cultivation Program of Longhua Hospital Affiliated to Shanghai University of Traditional Chinese Medicine (grant no. PY2022008).

## Conflict of interest

The authors declare that the research was conducted in the absence of any commercial or financial relationships that could be construed as a potential conflict of interest.

## Publisher’s note

All claims expressed in this article are solely those of the authors and do not necessarily represent those of their affiliated organizations, or those of the publisher, the editors and the reviewers. Any product that may be evaluated in this article, or claim that may be made by its manufacturer, is not guaranteed or endorsed by the publisher.

## Footnotes

^1^ https://www.crd.york.ac.uk/PROSPERO/display_record.php?RecordID=367811

## Supplementary material

SUPPLEMENTARY Table S1 Search strategy from database inception to April, 2023, for meta-analyses of randomized controlled trials.

SUPPLEMENTARY TABLE S2 Excluded studies with reasons from the search for meta-analyses of randomized controlled trials.

SUPPLEMENTARY TABLE S3 Summary of associations of NAC and outcomes with detail of GRADE assessment.

SUPPLEMENTARY TABLE S4 Summary of sensitivity analyses.

SUPPLEMENTARY FIGURE S1 Publication bias of NAC on CIN outcome. SE, standard error.

SUPPLEMENTARY FIGURE S2 Meta-analysis of studies examining the effect of NAC on CIN outcome based on OR. 95% CI, 95% confidence interval; IV, inverse variance.

SUPPLEMENTARY FIGURE S3 Meta-analysis of studies examining the effect of NAC on CIN outcome based on RR. 95% CI, 95% confidence interval; IV, inverse variance.

SUPPLEMENTARY FIGURE S4 Publication bias of NAC on Scr Levels. SE, standard error.

## Abbreviations

CIN: contrast-induced nephropathy
NAC: N-acetylcysteine
RCTs: randomized clinical studies
SCr: serum creatinine
AMSTAR-2: Assessment of Multiple Systematic Reviews, Version 2
OR: odds ratio
RR: relative risk
MD: mean deviation
SMD: standard mean deviation
