# Supplementary material for: Association of N-acetylcysteine use with contrast-induced nephropathy: an umbrella review of meta-analyses of randomized clinical trials
Source: Front Med (Lausanne). 2023 Sep 14;10:1235023. doi: 10.3389/fmed.2023.1235023 (PMC10543416; doi:10.3389/fmed.2023.1235023)
Supplement: SUPPLEMENTARY Table S1 — Search strategy from database inception to April, 2023, for meta-analyses of randomized controlled trials. [file Table_1.docx]

**Supplemental Table 1. Search Strategy from Database Inception to April, 2023, for Meta-Analyses of Randomized Controlled Trials.**

| **Database** | **Search term** | **Results** |
| --- | --- | --- |
| PubMed | ('Contrast Media' OR 'contrast medium' OR 'contrast material' OR 'contrast agent' OR 'contrast procedure' OR 'ontrast dye' OR 'contrast exposure' OR 'contrast-based' OR 'contrast induced' OR 'contrast imag' OR 'radiographic contrast radiocontrast' OR 'radio contrast ioxithalamate' OR 'meglumine' OR 'iopamidol' OR 'iohexol' OR 'iopromide' OR 'iotrolan' OR 'iodixanol' OR 'ioversol') AND ('nephropathy' OR 'kidney disease' OR 'kidney injury' OR 'kidney failure' OR 'renal failure' OR 'kidney insufficiency' OR 'renal insufficiency') AND ('acetylcysteine' OR 'n-acetyl-l-cysteine' OR 'n-acetyl cysteine' OR 'NAC' OR 'acetadote' OR 'fluimucil' OR 'mucomyst') AND ('systematic review' OR 'systematic literature review' OR 'meta-analysis' OR 'meta-analyses' OR 'meta-analysis' OR 'meta–analyses') | 182 |
| Cochrane Library | ('Contrast Media' OR 'contrast medium' OR 'contrast material' OR 'contrast agent' OR 'contrast procedure' OR 'ontrast dye' OR 'contrast exposure' OR 'contrast-based' OR 'contrast induced' OR 'contrast imag' OR 'radiographic contrast radiocontrast' OR 'radio contrast ioxithalamate' OR 'meglumine' OR 'iopamidol' OR 'iohexol' OR 'iopromide' OR 'iotrolan' OR 'iodixanol' OR 'ioversol') AND ('nephropathy' OR 'kidney disease' OR 'kidney injury' OR 'kidney failure' OR 'renal failure' OR 'kidney insufficiency' OR 'renal insufficiency') AND ('acetylcysteine' OR 'n-acetyl-l-cysteine' OR 'n-acetyl cysteine' OR 'NAC' OR 'acetadote' OR 'fluimucil' OR 'mucomyst') AND ('systematic review' OR 'systematic literature review' OR 'meta-analysis' OR 'meta-analyses' OR 'meta-analysis' OR 'meta–analyses') in Title Abstract Keyword - in Cochrane Reviews | 12 |
| EMBASE | ('Contrast Media' OR 'contrast medium' OR 'contrast material' OR 'contrast agent' OR 'contrast procedure' OR 'ontrast dye' OR 'contrast exposure' OR 'contrast-based' OR 'contrast induced' OR 'contrast imag' OR 'radiographic contrast radiocontrast' OR 'radio contrast ioxithalamate' OR 'meglumine' OR 'iopamidol' OR 'iohexol' OR 'iopromide' OR 'iotrolan' OR 'iodixanol' OR 'ioversol') AND ('nephropathy' OR 'kidney disease' OR 'kidney injury' OR 'kidney failure' OR 'renal failure' OR 'kidney insufficiency' OR 'renal insufficiency') AND ('acetylcysteine' OR 'n-acetyl-l-cysteine' OR 'n-acetyl cysteine' OR 'NAC' OR 'acetadote' OR 'fluimucil' OR 'mucomyst') AND ('systematic review' OR 'systematic literature review' OR 'meta-analysis' OR 'meta-analyses' OR 'meta-analysis' OR 'meta–analyses') | 137 |
| Web of science | ('Contrast Media' OR 'contrast medium' OR 'contrast material' OR 'contrast agent' OR 'contrast procedure' OR 'ontrast dye' OR 'contrast exposure' OR 'contrast-based' OR 'contrast induced' OR 'contrast imag' OR 'radiographic contrast radiocontrast' OR 'radio contrast ioxithalamate' OR 'meglumine' OR 'iopamidol' OR 'iohexol' OR 'iopromide' OR 'iotrolan' OR 'iodixanol' OR 'ioversol') AND ('nephropathy' OR 'kidney disease' OR 'kidney injury' OR 'kidney failure' OR 'renal failure' OR 'kidney insufficiency' OR 'renal insufficiency') AND ('acetylcysteine' OR 'n-acetyl-l-cysteine' OR 'n-acetyl cysteine' OR 'NAC' OR 'acetadote' OR 'fluimucil' OR 'mucomyst') AND ('systematic review' OR 'systematic literature review' OR 'meta-analysis' OR 'meta-analyses' OR 'meta-analysis' OR 'meta–analyses') in Title Abstract Keyword | 162 |
| **TOTAL** |  | 493 |
