# Supplementary material for: Association of N-acetylcysteine use with contrast-induced nephropathy: an umbrella review of meta-analyses of randomized clinical trials
Source: Front Med (Lausanne). 2023 Sep 14;10:1235023. doi: 10.3389/fmed.2023.1235023 (PMC10543416; doi:10.3389/fmed.2023.1235023)
Supplement: SUPPLEMENTARY TABLE S2 — Excluded studies with reasons from the search for meta-analyses of randomized controlled trials. [file Table_2.docx]

**Supplemental Table 2. Excluded Studies with Reasons from the Search for Meta-Analyses of Randomized Controlled Trials.**

| **Reasons for exclusion** | **References** |
| --- | --- |
| Not a meta-analysis  (n = 9) | 1. Weisbord SD, Palevsky PM. Radiocontrast-induced acute renal failure. *J Intensive Care Med*. Mar-Apr 2005;20(2):63-75. doi:10.1177/0885066604273503  2. Shalansky SJ, Vu T, Pate GE, Levin A, Humphries KH, Webb JG. N-acetylcysteine for prevention of radiographic contrast material-induced nephropathy: is the intravenous route best? *Pharmacotherapy*. Aug 2005;25(8):1095-103. doi:10.1592/phco.2005.25.8.1095  3. Hoffmann U, Banas B, Fischereder M, Kramer BK. N-acetylcysteine in the prevention of radiocontrast-induced nephropathy: clinical trials and end points. *Kidney Blood Press Res*. 2004;27(3):161-6. doi:10.1159/000079804  4. Ashworth A, Webb ST. Does the prophylactic administration of N-acetylcysteine prevent acute kidney injury following cardiac surgery? *Interact Cardiovasc Thorac Surg*. Sep 2010;11(3):303-8. doi:10.1510/icvts.2010.232413  5. investigator A. Acetylcysteine for prevention of renal outcomes in patients undergoing coronary and peripheral vascular angiography: main results from the randomized Acetylcysteine for Contrast-induced nephropathy Trial. *Circulation*. Sep 13 2011;124(11):1250-9. doi:10.1161/circulationaha.111.038943  6. Goldenberg I, Shechter M, Matetzky S, et al. Oral acetylcysteine as an adjunct to saline hydration for the prevention of contrast-induced nephropathy following coronary angiography. A randomized controlled trial and review of the current literature. *European heart journal*. Feb 2004;25(3):212-8. doi:10.1016/j.ehj.2003.11.011  7. Gawenda M, Möller A, Wassmer G, Brunkwall J. Prophylaxis of contrast-induced nephropathy with N-acetylcysteine. *Zentralbl Chir*. Jun 2007;132(3):227-31. N-Acetylcystein in der Prophylaxe der Kontrastmittel-induzierten Nephropathie. doi:10.1055/s-2007-960756  8. Izcovich A, Rada G. Should acetylcysteine be used to prevent contrast induced nephropathy? *Medwave*. Apr 15 2015;15(3):e6122. doi:10.5867/medwave.2015.03.6122 |
| Not intervention of interest  (n = 14) | 9. Khan SU, Khan MU, Rahman H, et al. A Bayesian network meta-analysis of preventive strategies for contrast-induced nephropathy after cardiac catheterization. *Cardiovasc Revasc Med*. Jan 2019;20(1):29-37. doi:10.1016/j.carrev.2018.06.005  10. Ahmed K, McVeigh T, Cerneviciute R, et al. Effectiveness of contrast-associated acute kidney injury prevention methods; a systematic review and network meta-analysis. *BMC Nephrol*. Nov 13 2018;19(1):323. doi:10.1186/s12882-018-1113-0  11. Ali-Hasan-Al-Saegh S, Mirhosseini SJ, Ghodratipour Z, et al. Strategies Preventing Contrast-Induced Nephropathy After Coronary Angiography: A Comprehensive Meta-Analysis and Systematic Review of 125 Randomized Controlled Trials. *Angiology*. May 2017;68(5):389-413. doi:10.1177/0003319716661445  12. Dong M, Jiao Z, Liu T, Guo F, Li G. Effect of administration route on the renal safety of contrast agents: a meta-analysis of randomized controlled trials. *J Nephrol*. May-Jun 2012;25(3):290-301. doi:10.5301/jn.5000067  13. Giacoppo D, Gargiulo G, Buccheri S, et al. Preventive Strategies for Contrast-Induced Acute Kidney Injury in Patients Undergoing Percutaneous Coronary Procedures: Evidence From a Hierarchical Bayesian Network Meta-Analysis of 124 Trials and 28 240 Patients. *Circ Cardiovasc Interv*. May 2017;10(5)doi:10.1161/circinterventions.116.004383  14. Kelly AM, Dwamena B, Cronin P, Bernstein SJ, Carlos RC. Meta-analysis: effectiveness of drugs for preventing contrast-induced nephropathy. *Ann Intern Med*. Feb 19 2008;148(4):284-94. doi:10.7326/0003-4819-148-4-200802190-00007  15. Ma WQ, Zhao Y, Wang Y, Han XQ, Zhu Y, Liu NF. Comparative efficacy of pharmacological interventions for contrast-induced nephropathy prevention after coronary angiography: a network meta-analysis from randomized trials. *Int Urol Nephrol*. Jun 2018;50(6):1085-1095. doi:10.1007/s11255-018-1814-0  16. Mattathil S, Ghumman S, Weinerman J, Prasad A. Use of the RenalGuard system to prevent contrast-induced AKI: A meta-analysis. *J Interv Cardiol*. Oct 2017;30(5):480-487. doi:10.1111/joic.12417  17. Putzu A, Boscolo Berto M, Belletti A, et al. Prevention of Contrast-Induced Acute Kidney Injury by Furosemide With Matched Hydration in Patients Undergoing Interventional Procedures: A Systematic Review and Meta-Analysis of Randomized Trials. *JACC Cardiovasc Interv*. Feb 27 2017;10(4):355-363. doi:10.1016/j.jcin.2016.11.006  18. Su X, Xie X, Liu L, et al. Comparative Effectiveness of 12 Treatment Strategies for Preventing Contrast-Induced Acute Kidney Injury: A Systematic Review and Bayesian Network Meta-analysis. *Am J Kidney Dis*. Jan 2017;69(1):69-77. doi:10.1053/j.ajkd.2016.07.033  19. Kwok CS, Pang CL, Yeong JK, Loke YK. Measures used to treat contrast-induced nephropathy: overview of reviews. *Br J Radiol*. Jan 2013;86(1021):20120272. doi:10.1259/bjr.20120272  20. Walker H, Guthrie GD, Lambourg E, et al. Systematic review and meta-analysis of prophylaxis use with intravenous contrast exposure to prevent contrast-induced nephropathy. *Eur J Radiol*. Aug 2022;153:110368. doi:10.1016/j.ejrad.2022.110368  21. Zoungas S, Ninomiya T, Huxley R, et al. Systematic review: sodium bicarbonate treatment regimens for the prevention of contrast-induced nephropathy. *Ann Intern Med*. Nov 3 2009;151(9):631-8. doi:10.7326/0003-4819-151-9-200911030-00008  22. Ali-Hasan-Al-Saegh S, Mirhosseini SJ, Ghodratipour Z, et al. Protective effects of anti-oxidant supplementations on contrast-induced nephropathy after coronary angiography: an updated and comprehensive meta-analysis and systematic review. *Kardiol Pol*. 2016;74(7):610-26. doi:10.5603/KP.a2016.0007 |
| Not a meta-analysis with the largest data set  (n = 6) | 23. Birck R, Krzossok S, Markowetz F, Schnulle P, van der Woude FJ, Braun C. Acetylcysteine for prevention of contrast nephropathy: meta-analysis. *Lancet*. Aug 23 2003;362(9384):598-603. doi:10.1016/S0140-6736(03)14189-X  24. Kang X, Hu DY, Li CB, Ai ZS, Peng A. N-acetylcysteine for the prevention of contrast-induced nephropathy in patients with pre-existing renal insufficiency or diabetes: a systematic review and meta-analysis. *Ren Fail*. Nov 2015;37(10):297-303. doi:10.3109/0886022x.2015.1012985  25. Misra D, Leibowitz K, Gowda RM, Shapiro M, Khan IA. Role of N-acetylcysteine in prevention of contrast-induced nephropathy after cardiovascular procedures: a meta-analysis. *Clinical cardiology*. Nov 2004;27(11):607-10. doi:10.1002/clc.4960271106  26. O'Sullivan S, Healy DA, Moloney MC, Grace PA, Walsh SR. The role of N--acetylcysteine in the prevention of contrast-induced nephropathy in patients undergoing peripheral angiography: a structured review and meta-analysis. *Angiology*. Nov 2013;64(8):576-82. doi:10.1177/0003319712467223  27. Sun Z, Fu Q, Cao L, Jin W, Cheng L, Li Z. Intravenous N-acetylcysteine for prevention of contrast-induced nephropathy: a meta-analysis of randomized, controlled trials. *PLoS One*. 2013;8(1):e55124. doi:10.1371/journal.pone.0055124  28. Zhaodong G, Jin L, Li L, et al. Effect of N-acetylcysteine on prevention of contrast-associated acute kidney injury in patients with STEMI undergoing primary percutaneous coronary intervention: a systematic review and meta-analysis of randomised controlled trials. *BMJ open*. 2020;10(10)  29. Liu R, Nair D, Ix J, Moore DH, Bent S. N-acetylcysteine for the prevention of contrast-induced nephropathy. A systematic review and meta-analysis. J Gen Intern Med. Feb 2005;20(2):193-200. doi:10.1111/j.1525-1497.2005.30323.x |
| Acetylcysteine combines with other drugs  (n = 3) | 30. Zhao SJ, Zhong ZS, Qi GX, Tian W. The efficacy of N-acetylcysteine plus sodium bicarbonate in the prevention of contrast-induced nephropathy after cardiac catheterization and percutaneous coronary intervention: A meta-analysis of randomized controlled trials. International journal of cardiology. Oct 15 2016;221:251-9. doi:10.1016/j.ijcard.2016.07.086  31. Feng Y, Huang X, Li L, Chen Z. N-acetylcysteine versus ascorbic acid or N-acetylcysteine plus ascorbic acid in preventing contrast-induced nephropathy: A meta-analysis. Nephrology (Carlton). Jun 2018;23(6):530-538. doi:10.1111/nep.13068  32. Brown JR, Block CA, Malenka DJ, O'Connor GT, Schoolwerth AC, Thompson CA. Sodium bicarbonate plus N-acetylcysteine prophylaxis: a meta-analysis. JACC Cardiovasc Interv. Nov 2009;2(11):1116-24. doi:10.1016/j.jcin.2009.07.015 |
| Case study  (n = 2) | 33. Bagshaw SM, McAlister FA, Manns BJ, Ghali WA. Acetylcysteine in the prevention of contrast-induced nephropathy: a case study of the pitfalls in the evolution of evidence. Archives of internal medicine. Jan 23 2006;166(2):161-6. doi:10.1001/archinte.166.2.161  34. Biondi-Zoccai GG, Lotrionte M, Abbate A, et al. Compliance with QUOROM and quality of reporting of overlapping meta-analyses on the role of acetylcysteine in the prevention of contrast associated nephropathy: case study. BMJ. Jan 28 2006;332(7535):202-9. doi:10.1136/bmj.38693.516782.7C |
