# Supplementary material for: Association of N-acetylcysteine use with contrast-induced nephropathy: an umbrella review of meta-analyses of randomized clinical trials
Source: Front Med (Lausanne). 2023 Sep 14;10:1235023. doi: 10.3389/fmed.2023.1235023 (PMC10543416; doi:10.3389/fmed.2023.1235023)
Supplement: SUPPLEMENTARY TABLE S3 — Summary of associations of NAC and outcomes with detail of GRADE assessment. [file Table_3.docx]

**Supplemental Table 3. Summary of Associations of NAC and Outcomes with Detail of GRADE Assessment.**

| **Source** | **Outcome** | **No. of Participants** | **GRADE evidence [Not serious (NS), serious (S), very serious (VS), not clear (NC)]** | | | | | |
| --- | --- | --- | --- | --- | --- | --- | --- | --- |
|  |  |  | **Risk of bias** | **Inconsistency** | **Indirectness** | **Imprecision** | **publication bias** | **Overall certainty of evidence** |
| Bagshaw  2004 | CIN incidence | 1365 | NS | NS | NS | NS | NC | High |
| Bagshaw  2004 | change in Scr | 623 | NS | NS | NS | NS | NC | High |
| Feng  2018 | CIN incidence | 683 | NS | S | NS | S | NC | Moderate |
| Feng  2018 | change in Scr | 663 | NS | NS | NS | NS | NC | High |
| Gonzales  2007 | CIN incidence | 2746 | NC | VS | NS | VS | NC | Low |
| Gonzales  2007 | requirement for dialysis | 2746 | NC | VS | NS | VS | NC | Low |
| Li  2017 | CIN incidence | 4514 | NS | NS | NS | S | NC | Moderate |
| Loomba  2016 | CIN incidence | 5199 | NS | S | NS | NS | S | Moderate |
| Loomba  2016 | requirement for dialysis | 3585 | NS | S | NS | S | S | Moderate |
| Loomba  2016 | mortality | 453 | NS | NS | NS | NS | NC | High |
| Loomba  2016 | change in Scr | 1868 | NS | VS | NS | VS | NS | Low |
| Magner  2022 | CIN incidence | 32235 | NS | NS | NS | NS | NC | Moderate |
| Trivedi  2009 | CIN incidence | 1677 | NS | NS | NS | S | NC | Moderate |
| Wang  2016 | CIN incidence | 6554 | NS | NS | NS | NS | S | Moderate |
| Wu  2013 | CIN incidence | 485 | VS | NS | NS | NS | VS | Low |
| Wu  2013 | change in Scr | 233 | VS | NS | NS | NS | VS | Low |
| Xie  2021 | CIN incidence | 18592 | NS | VS | VS | S | NS | Moderate |
| Xie  2021 | change in Scr | 1318 | NS | NS | NS | S | NS | Moderate |
| Xu  2016 | CIN incidence | 11481 | S | NS | NS | NS | S | Moderate |
| Zagler  2006 | CIN incidence | 1892 | NS | NS | NS | NS | NC | High |
