# Supplementary material for: Association of N-acetylcysteine use with contrast-induced nephropathy: an umbrella review of meta-analyses of randomized clinical trials
Source: Front Med (Lausanne). 2023 Sep 14;10:1235023. doi: 10.3389/fmed.2023.1235023 (PMC10543416; doi:10.3389/fmed.2023.1235023)
Supplement: SUPPLEMENTARY TABLE S4 — Summary of sensitivity analyses. [file Table_4.docx]

**Supplemental Table 4. Summary of Sensitivity Analyses.**

| **Source** | **Population** | **Metric** | **Primary analysis** | | | **Sensitivity analysis: Excluding studies with high risk of bias** | | | **Sensitivity analysis: Excluding studies with small sample size (25th percentile)** | | |
| --- | --- | --- | --- | --- | --- | --- | --- | --- | --- | --- | --- |
|  |  |  | **No. of studies** | **Effect size**  **(95% CI)** | **GRADE** | **No. of studies** | **Effect size**  **(95% CI)** | **GRADE** | **No. of studies** | **Effect size**  **(95% CI)** | **GRADE** |
| **CIN incidence** | | | | | | | | | | | |
| Bagshaw  2004 | CIN patients undergoing intravascular angiography | OR | 14 | 0.54 (0.32 to 0.91) | High | 9 | 0.58 (0.29 to 1.15) | High | 11 | 0.69 (0.41 to 1.14) | High |
| Feng  2018 | CIN patients undergoing contrast administration | RR | 3 | 0.70 (0.28 to 1.74) | Moderate | 3 | 0.70 (0.28 to 1.74) | Moderate | Remaining studies are not enough to conduct meta-analysis | | |
| Gonzales  2007 | CIN (CT, LHC, PCI, PA) | OR | 22 | 0.64 (0.50 to 0.82) | Low | 10 | 0.44 (0.31 to 0.63) | Moderate | 16 | 0.65 (0.49 to 0.86) | Low |
| Li  2017 | CIN patients undergoing coronary angioplasty | RR | 19 | 0.84 (0.65 to 1.10) | Moderate | 18 | 0.81 (0.61 to 1.08) | Moderate | 14 | 0.87 (0.66 to 1.15) | Moderate |
| Loomba  2016 | CIN patients undergoing  peripheral vascular or coronary angiography | OR | 23 | 0.70 (0.51 to 0.95) | Moderate | 23* | 0.70 (0.51 to 0.95) | Moderate | 17 | 0.78 (0.58 to 1.06) | Moderate |
| Magner  2022 | CIN | OR | 101 | 0.72 (0.63 to 0.82) | Moderate | 101* | 0.72 (0.63 to 0.82) | Moderate | No detailed data of the RCT | | |
| Trivedi  2009 | CIN | OR | 16 | 0.46 (0.33 to0.63) | Moderate | 16* | 0.46 (0.33 to0.63) | Moderate | No detailed data of the RCT | | |
| Wang  2016 | CIN patients undergoing CAG with or  without PCI | OR | 43 | 0.66 (0.53 to 0.83) | Moderate | 43* | 0.66 (0.53 to 0.83) | Moderate | 32 | 0.72 (0.57 to 0.92) | Moderate |
| Wu  2013 | CIN patients undergoing contrast enhanced CT | RR | 6 | 0.34 (0.18 to 0.64) | Low | 4 | 0.41 (0.2 to 0.81) | Moderate | 4 | 0.33 (0.16 to 0.67) | Low |
| Xie  2021 | CIN | RR | 57 | 0.78 (0.68 to 0.90) | Moderate | 42 | 0.85 (0.74 to 0.98) | Moderate | 43 | 0.81 (0.70 to 0.92) | Moderate |
| Xu  2016 | CIN | RR | 66 | 0.76 (0.66 to 0.88) | Moderate | 55 | 0.77 (0.66 to 0.89) | Moderate | 43 | 0.82 (0.71 to 0.95) | Moderate |
| Zagler  2006 | CIN | RR | 13 | 0.68 (0.46 to 1.02) | High | 13* | 0.68 (0.46 to 1.02) | High | 10 | 0.67 (0.43 to 1.05) | High |
|  | | | | | | | | | | | |
| **Change in Scr** | | | | | | | | | | | |
| Bagshaw  2004 | CIN patients undergoing intravascular angiography | MD | 8 | -1.8 (-8.9 to 5.2) | High | No detailed data of the standard deviation | | | No detailed data of the standard deviation | | |
| Feng  2018 | CIN patients undergoing contrast administration | MD | 4 | -0.05 (-0.08 to 0.04) | High | 4 | -0.05 (-0.08 to 0.04) | High | 3 | -0.06 (-0.10 to-0.02) | High |
| Loomba  2016 | CIN patients undergoing  peripheral vascular or coronary angiography | MD | 16 | -0.10 (-0.22 to 0.03) | Low | 16* | -0.10 (-0.22 to 0.03) | Low | 12 | -0.09 (-0.25 to 0.07) | Low |
| Wu  2013 | CIN patients undergoing contrast enhanced CT | MD | 4 | -0.22 (-0.41 to -0.03) | Low | 2 | -0.08 (-0.24 to 0.07) | Moderate | 3 | -0.30 (-0.54 to -0.07) | Low |
| Xie  2021 | CIN | SMD | 11 | -0.53 (-0.93 to -0.12) | Moderate | 9 | -0.59 (-1.07 to -0.10) | Moderate | 8 | -0.20 (-0.4 to 0.06) | Moderate |
|  | | | | | | | | | | | |
| **Requirement for dialysis** | | | | | | | | | | | |
| Gonzales  2007 | CIN (CT, LHC, PCI, PA) | RR | 22 | 1.42 (0.46 to 4.39) | Low | No detailed data | | | No detailed data | | |
| Loomba  2016 | CIN patients undergoing  peripheral vascular or coronary angiography | OR | 7 | 1.66 (0.67 to 4.12) | Moderate | 7* | 1.66 (0.67 to 4.12) | Moderate | 5 | 1.68 (0.59 to 4.83) | Moderate |
|  | | | | | | | | | | | |
| **Mortality** | | | | | | | | | | | |
| Loomba  2016 | CIN patients undergoing  peripheral vascular or coronary angiography | OR | 7 | 0.85 (0.56 to 1.29) | High | 7* | 0.85 (0.56 to 1.29) | High | 5 | 0.94 (0.60 to 1.46) | High |

*, no evaluation of risk of bias; CT, computed tomography; LHC, left heart catheterization; PCI, percutaneous coronary intervention; PA, peripheral angiography; ST segment elevation myocardial infarction (STEMI); PPCI, primary percutaneous coronary intervention; NAC, N-acetylcysteine; NaHCO3; NS,saline solution; CAG, coronary angiography.
