# Supplementary figures and images for: Association of N-acetylcysteine use with contrast-induced nephropathy: an umbrella review of meta-analyses of randomized clinical trials
Source: Front Med (Lausanne). 2023 Sep 14;10:1235023. doi: 10.3389/fmed.2023.1235023 (PMC10543416; doi:10.3389/fmed.2023.1235023)

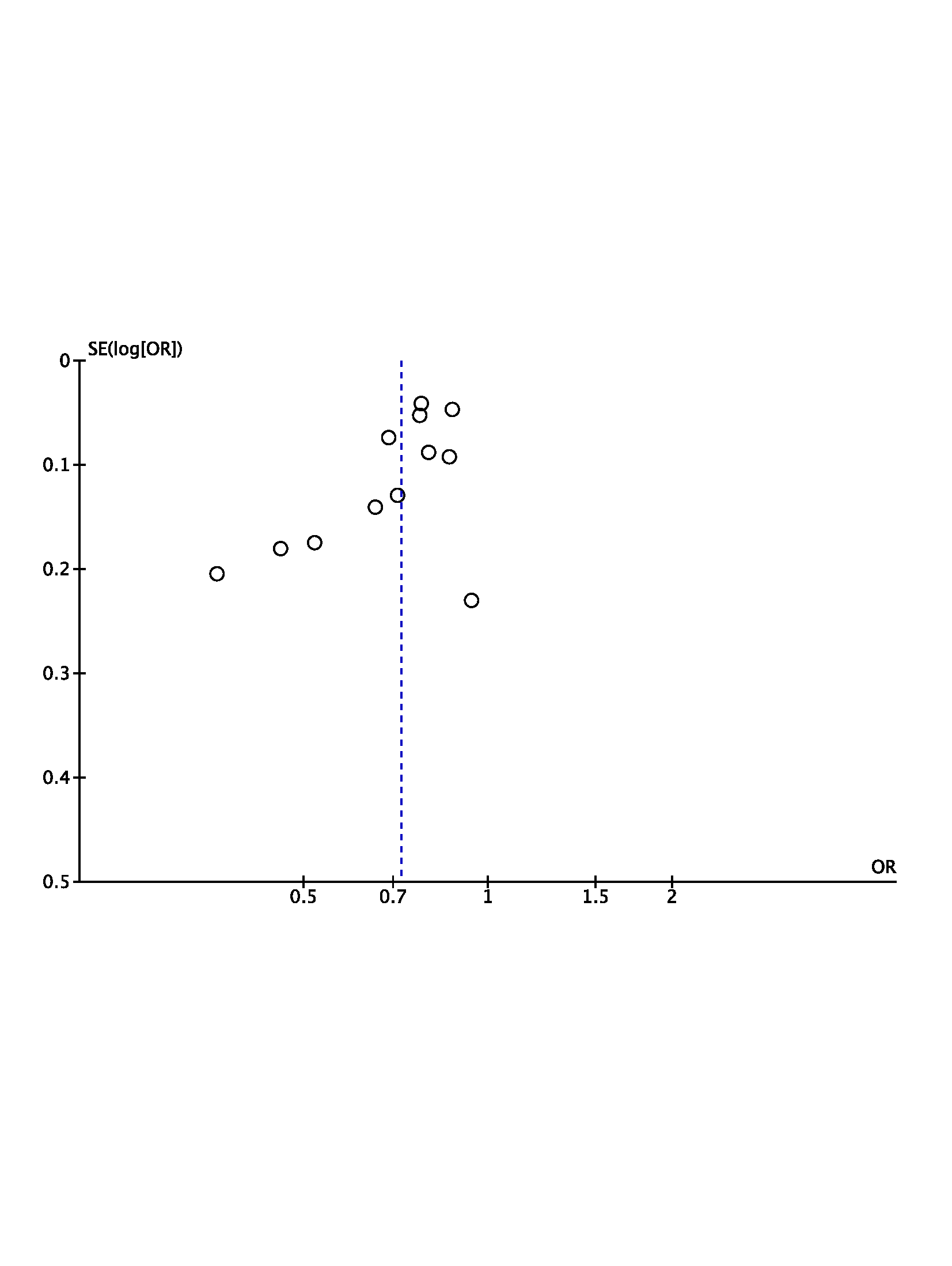

Supplement: SUPPLEMENTARY FIGURE S1 — Publication bias of NAC on CIN outcome. SE, standard error. [file Image_1.TIFF]

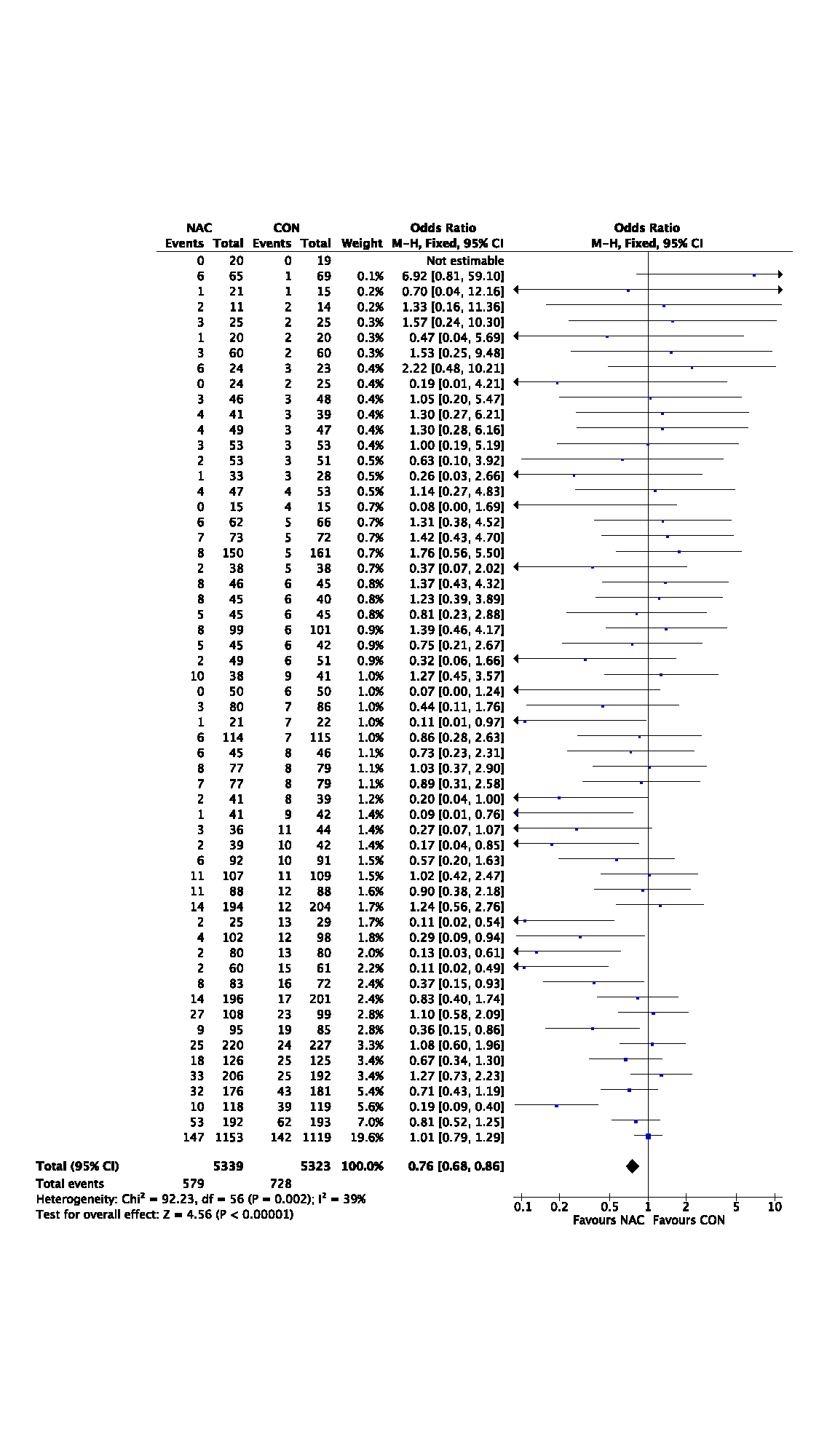

Supplement: SUPPLEMENTARY FIGURE S2 — Meta-analysis of studies examining the effect of NAC on CIN outcome based on OR. 95% CI, 95% confidence interval; IV, inverse variance. [file Image_2.TIFF]

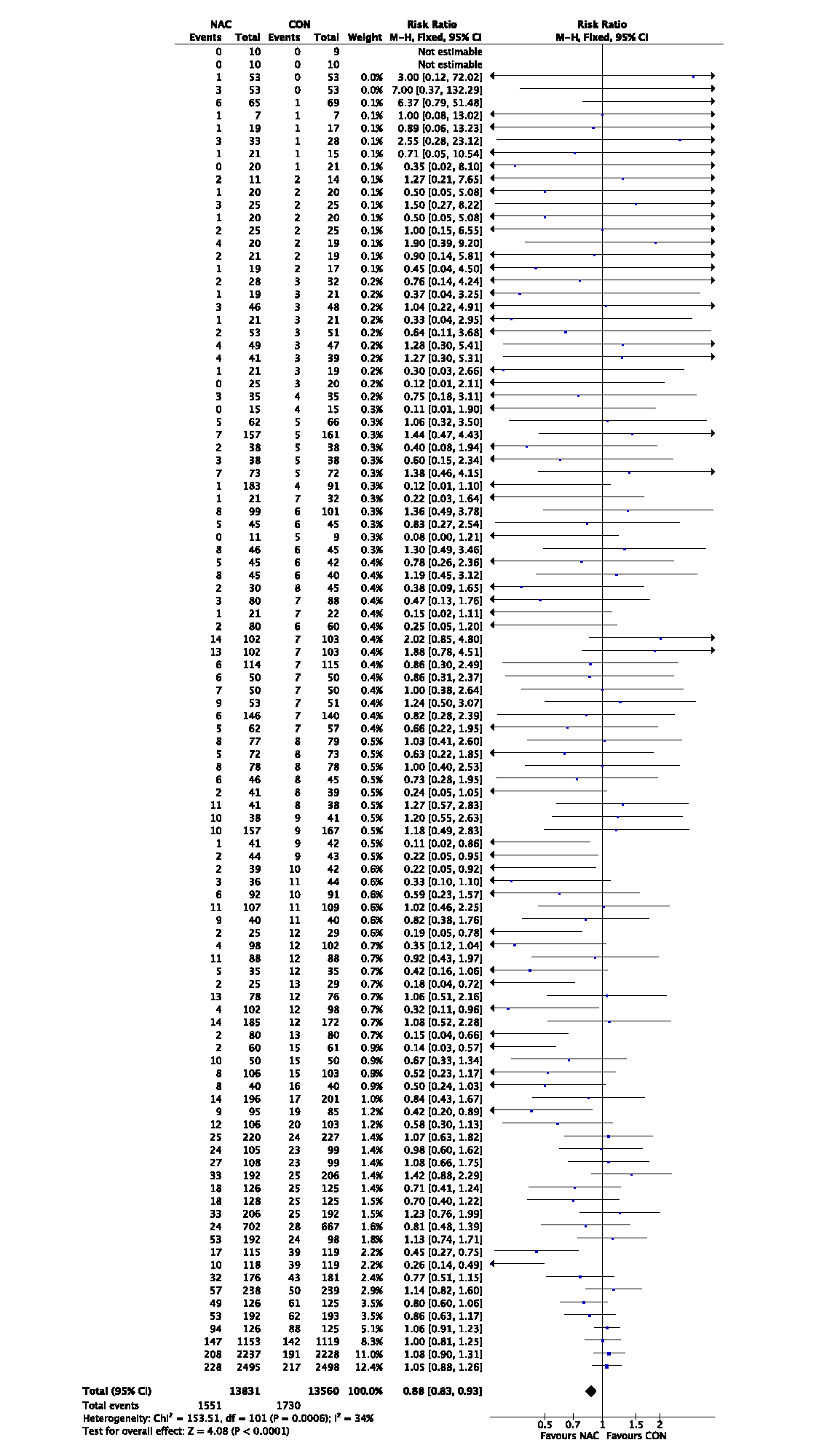

Supplement: SUPPLEMENTARY FIGURE S3 — Meta-analysis of studies examining the effect of NAC on CIN outcome based on RR. 95% CI, 95% confidence interval; IV, inverse variance. [file Image_3.TIFF]

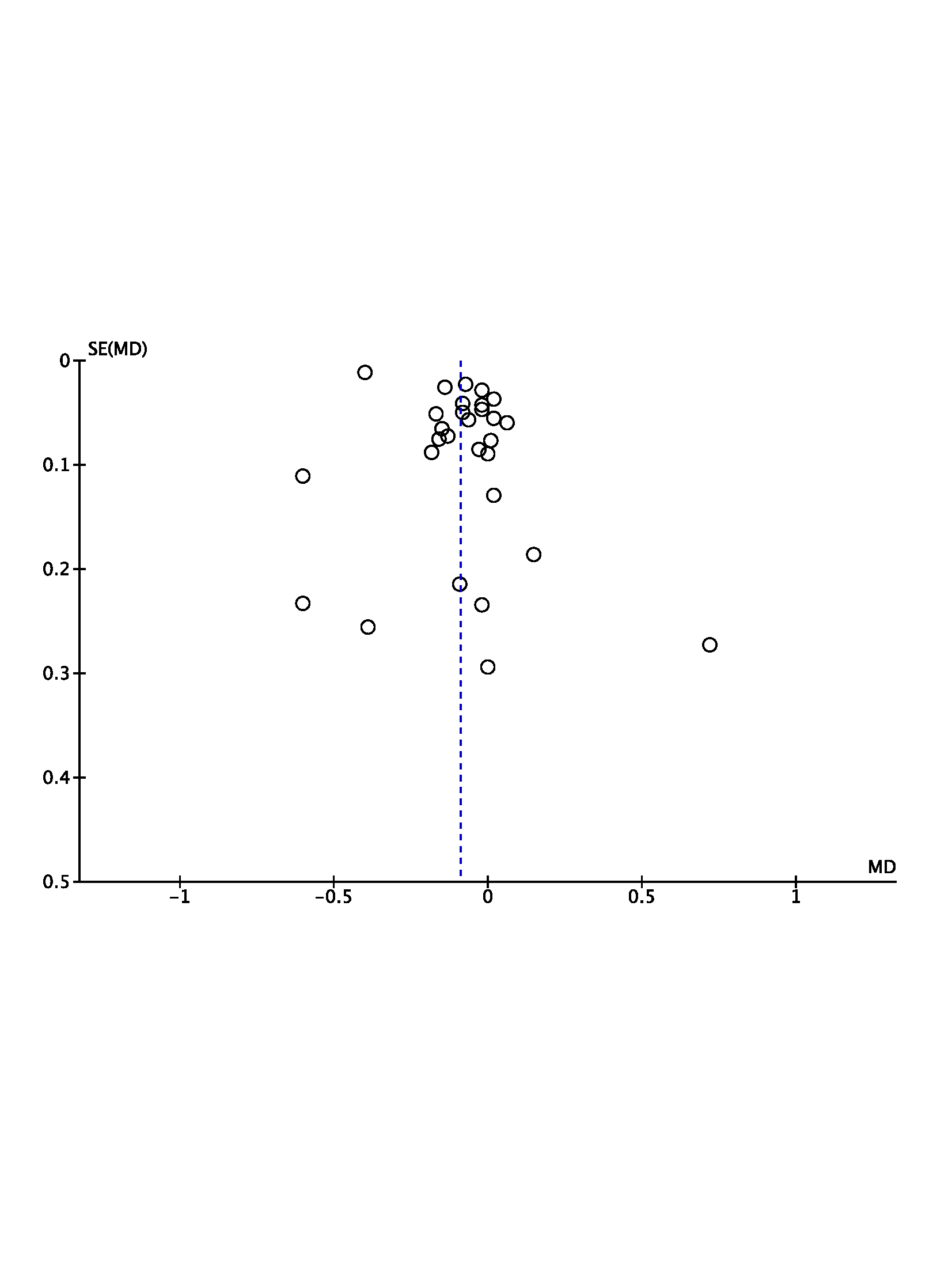

Supplement: SUPPLEMENTARY FIGURE S4 — Publication bias of NAC on Scr Levels. SE, standard error. [file Image_4.TIFF]
